# Supplementary material for: Are carbon emissions trading and green financial instruments synergistic? -Comprehensive quantitative research based on content analysis
Source: PLoS One. 2024 Mar 7;19(3):e0298601. doi: 10.1371/journal.pone.0298601 (PMC10919601; doi:10.1371/journal.pone.0298601)
Supplement: S1 Data — (ZIP) [file pone.0298601.s001.zip › Data/地级市Z_F工作报告.zip!/╣π╢1⁄2/╗▌╓▌╩╨/└┤╫╘╣1⁄2╓┌║┼í╛┬φ┐╦╩2╛▌═°í┐_2016.docx]

各位代表：

　　现在，我代表市人民政府向大会报告工作，请予审议，并请政协各位委员和其他列席人员提出意见。

　　“十二五”时期工作回顾

　　“十二五”时期是我市跨越发展的五年。面对复杂多变的发展形势，市政府在省委、省政府和市委的正确领导下，在市人大及其常委会和市政协的监督支持下，全面贯彻党的十八大和十八届三中、四中、五中全会精神，深入学习贯彻习近平总书记系列重要讲话精神，紧紧围绕尽快进入珠三角第二梯队总目标，坚持稳中求进、好中求快、改革创新、率先跨越，大力实施珠三角改革发展规划纲要，加快推进“六大计划”，统筹做好稳增长、促改革、调结构、惠民生、防风险各项工作，顺利完成了“十二五”主要目标任务。

　　“十二五”期间，全市地区生产总值相继突破2000亿元、3000亿元，年均增长12%，2015年达3140亿元，增长9%，增速居全省第2位。人均GDP突破1万美元，年均增长10.7%，2015年达6.62万元，增长8.4%。地方一般公共预算收入相继突破200亿元、300亿元，年均增长21%，2015年达339.99亿元，增长13.1%。经济总量和财政收入跃居全省第五位，实现了争先进位。立体交通大格局基本形成，先后迈进“高铁时代”“航空时代”，又将迎来“城轨时代”。成功夺取全国文明城市“三连冠”，成功创建国家森林城市，成功列入国家历史文化名城，宜居宜业宜游的城市品质大幅提升，经济社会发展取得新的重大成就。

　　五年来，我们全力推动经济持续健康较快发展，综合实力显著增强。面对持续加大的经济下行压力，始终把稳增长作为首要任务，主动作为、综合施策，促进经济稳中有进、稳中提质。充分发挥项目带动效应，实施重点项目455宗，建成投产220宗，2013年以来新立项亿元以上项目动工建设647宗，累计固定资产投资7105.8亿元，是 “十一五”的2.3倍。2015年，固定资产投资1863.9亿元，增长16%。着力扩大内外需求，成功举办4场惠州产品海外展览会、8场国内展销会、4届云博会、3届中国手机创新周活动，累计签订销售贸易合同2238.9亿元。房地产、汽车等大宗消费稳步上升，电子商务、信息消费、旅游休闲等成为消费新增长点。累计社会消费品零售总额4336.2亿元，是“十一五”的2倍。2015年，社会消费品零售总额1070.7亿元，增长10.5%。积极推动外经贸发展，新引进外资项目1584宗，实际利用外资82亿美元，其中世界500强企业投资项目6宗。对外贸易依存度降至107.5%，比“十一五”末下降26.5个百分点。“委托设计+自主品牌”出口占加工贸易出口比重从41.3%提高到69%。累计进出口总额2594.7亿美元，是“十一五”的1.9倍。2015年，进出口总额3376.1亿元，下降7.5%。大力实施“企业成长计划”，新培育千亿元企业2家，新增上市企业4家、新三板挂牌企业12家、天交所挂牌企业19家。累计在境外设立企业72家，对外投资3.4亿美元。扶持中小企业发展，累计完成“个转企”1614家、“小升规”912家，新增注册商标12587件、驰名商标18件。制定实施“惠28条”“惠42条”，民营经济增加值占GDP比重42.7%，五年提高7.2个百分点。

　　五年来，我们坚定不移深化改革，体制机制更有活力。重点领域和关键环节改革取得突破，20项国家级和54项省级改革试点有序推进，获评中国改革最具投资潜力城市。行政体制改革成效明显。实施“三张清单”管理制度，行政审批事项减少67%，行政许可事项减少43%。市政府工作部门由35个精简为32个，食品药品、工商、质监部门调整为地方政府管理，市水投、交投、城投、港投集团相继挂牌运作。公务用车制度改革全面完成。事业单位分类改革基本完成。政府全口径预算管理启动实施。政府公共服务满意度连续四年居全省第一。经济体制改革成效明显。实行商事登记制度改革，投资项目审批提速80%，2015年市场主体37.3万户，五年新增21万户。公共资源交易体制改革获评全国行政服务大厅十佳典型案例。网上办事大厅实现镇街、村居全覆盖。率先在全省建成市县一体化“中介超市”，获评全省政府治理创新优秀案例。城乡综合改革成效明显。在全省率先开展基本公共服务均等化综合改革，提前两年实现国家基本公共服务“十二五”目标。入选国家新型城镇化综合试点、国家智慧城市试点，成为首批国家信息消费试点城市。农村土地承包经营权确权登记颁证工作顺利推进。建成农村产权流转管理市级服务平台。探索开展集体经营性建设用地使用权流转。实施不动产统一登记。社会治理体制改革成效明显。“两建”考核连续3年综合排名全省前列。全国仲裁“两化”试点和反走私诚信体系建设试点工作深入推进。村（居）委“法制副主任”制度实现全覆盖，经验全省推广。实行社会组织直接登记制度，2015年每万人社会组织数6.87个，每万人持证社工人数5.45个。获评全国首批社会治理创新优秀市。

　　五年来，我们深入推进转型升级，产业结构进一步优化。紧紧围绕转变经济发展方式这条主线，大力实施“产业培育计划”，推动产业向高端集聚发展。三次产业结构由5.9∶58.6∶35.5调整为4.8∶55∶40.2。2015年，规模以上先进制造业和高技术制造业增加值占规模以上工业比重分别为59.2%和43.6%，达到全省先进水平。石油化工、电子信息产业集群发展。中海油乐金ABS、TCL移动互联网智能终端等一批项目建成投产，中海油惠炼二期、信利AMOLED一期等重大项目顺利推进，2015年石油化工、电子信息两大支柱产业增加值占规模以上工业比重达52.1%。汽车和新能源产业加快发展。东风本田汽车一体化项目（一期）、比亚迪锂电池项目建成投产，汽车零部件产业园列入省重点支持对象。广东太平岭核电项目启动，风力、太阳能光伏发电等新能源产业稳步推进。现代服务业优质发展。罗浮山成功创建国家5A级景区，南昆山入选国家生态旅游示范区，新增4A级景区2家。2015年，接待旅游总人数4076.8万人次，旅游总收入330.2亿元，分别是五年前的1.6倍、2.3倍。累计组建或引进银行机构15家、证券保险机构32家，县区农信社全部改制为农商银行。2015年末，金融机构本外币存贷款余额分别为3836.1亿元、2701.6亿元，存贷比达70.4%，五年提高11.8个百分点。特色农业稳步发展。耕地保护走在全省前列，基本农田保有量195.94万亩，建成标准农田100万亩、现代农业示范园6个，“十二大”特色农产品种植面积达214.5万亩。成功举办两届农博会，培育农业龙头企业260家、专业合作社1856家，农业科技贡献率达62.5%。

　　五年来，我们突出抓好创新驱动，经济增长内生动力不断提升。坚持把创新作为引领发展的第一动力，大力实施创新驱动发展战略，“1+6+N”创新政策红利加快释放。创新投入持续增长。全市财政一般预算科技支出累计达55.8亿元，全社会研发经费支出占地区生产总值的比重由1.11%提高到2.4%。设立创投基金11家，建立中小微企业贷款风险补偿基金，成立首家科技银行。2015年工业技改投资332.2亿元，五年增长6.4倍。创新能力持续增强。2015年高新技术企业、工程技术中心分别达255家、203家，五年分别增长1.6倍、2.1倍，技术自给率达70%。建成新型研发机构4家、在建9家，建成孵化器10家，其中国家级3家。创新要素加速集聚。大力推动协同创新，实施产学研项目523项，组建产学研联盟8个、院士工作站3个、产学研示范基地7个，荣获中国产学研合作促进奖。深入实施“人才双高计划”，加快打造创新创业人才高地，累计引进创新团队18个、领军人才38名。创新成果不断涌现。获国家科技进步二等奖1项、省科技进步二等奖3项、三等奖17项，中国专利金奖2项、优秀奖6项，2015年专利申请量和授权量分别达21408件、9797件，五年分别增长6.29倍、5.14倍，获评国家知识产权示范市，连续五次获评全国科技进步先进市。

　　五年来，我们着力统筹城乡一体化，城乡区域发展更加协调。围绕国家统筹城乡协调发展综合改革试点，深入实施 “平台构建计划”“县区赶超计划”，推动城乡区域一体化。城市建设扩容提质。编制实施主体功能区规划，市中心城区控制性详细规划基本实现全覆盖。打通47条市区连接路，“一城三组团”的辐射带动能力进一步增强，城市建成区面积扩大54平方公里，达320平方公里，常住人口城镇化率由61.84%提高到68.15%。智慧城市建设稳步推进，智慧政务全面实施，数字城管系统投入运行，无线宽带网络覆盖率、互联网普及率分别达75.7%、73.9%。清理闲置土地2396.9亩，实施“三旧”改造3922亩。“两违”整治深入推进，城市管理水平进一步提升。平台建设加快构建。环大亚湾新区上升为省重大区域发展平台，三大起步区35宗重点项目完成投资258亿元。仲恺高新区纳入珠三角国家自主创新示范区。潼湖生态智慧区完成总体规划编制，列入省重大创新平台。县域经济实力明显增强。七个县区人均GDP全部超过全国平均水平，县域经济总量占全市比重由35.2%提高到39%。博罗全国县域经济百强县地位更加稳固，博罗、惠东进入全省县域综合竞争力前三甲，龙门县域旅游综合竞争力连续五年居全省十强。基础设施水平全面跃升。厦深高铁通车，惠州机场通航，莞惠城轨惠州段建成。7条高速公路建成，实现县县通高速，新增高速公路208公里，通车总里程达587公里，居全省第三。国际集装箱码头投入使用，新增5万吨深水泊位2个，惠州港吞吐能力突破亿吨。完成国省干线公路改造建设554公里、农村公路硬底化4433公里。建成江海堤围113公里，治理河道146公里，完成20宗水库除险加固。变电站规模达139座，新增输电线路1867千米。建成天然气管道248.6公里，油气管道达514.6公里。两轮扶贫“双到”任务圆满完成。累计投入11亿元，131个省重点帮扶村年集体经济收入平均10万元以上，8.4万贫困人口全部脱贫。完成105宗农村饮水安全工程和74宗省级贫困村通自来水工程，解决了28.8万农村群众饮水问题。

　　五年来，我们大力推动绿色低碳发展，生态优势日益凸显。在全省率先出台低碳生态规划，连续8年全省环保责任考核优秀等级，连续三次获评中国最美丽城市，连续四次获评中国十佳宜居城市。空气质量不断改善，2015年排名全国74个重点监测城市第三，市区空气质量优良天数比例达97.5%。国家生态文明建设示范市创建工作扎实有效。成功创建国家级生态镇6个、省级生态镇53个、生态村748个。龙门县获评全省首个省级生态县。新建城镇污水处理厂48座、截污管网800公里，新增日处理能力64.9万吨，城镇生活污水处理率96%。新一轮绿化惠州大行动成效明显。新增生态景观林带783.4公里、碳汇森林50.4万亩，新建湿地公园4个、森林公园32个，森林覆盖率由59.6%提高到62.34%，城市人均公园绿地面积由11.01平方米提高到17.75平方米。全国水生态文明城市建设试点稳步推进。完成淡水河、潼湖流域污染整治阶段性任务，东江干流惠州段保持国家二类水质标准，城市饮用水源达标率100%。考洲洋美丽海湾建设进展顺利，近岸海域水质全省最优，成功创建国家级海洋生态文明示范区。实施市区河涌整治总体规划，打造金山河、青年河水环境综合整治样板工程，金山河水清岸绿工程获中国人居环境范例奖。“美丽乡村·三大行动”深入开展。建成农村污水处理设施477个、垃圾收集点2万多个、森林村庄285个，配备农村保洁员9333名；博罗县生活垃圾焚烧发电厂建成投产，惠阳区、惠东县垃圾焚烧发电厂顺利推进，城乡生活垃圾无害化处理率达95%，农村生产生活环境大幅改善。节能减排成效突出。清退重污染项目177宗，完成减排项目269宗。淘汰黄标车和老旧车辆4.68万辆，推广应用新能源汽车超2000辆。预计万元GDP能耗五年累计下降17%，化学需氧量和氨氮、氮氧化物排放量分别下降24.8%、22.5%、16%，全面完成省下达污染减排任务。

　　五年来，我们加快发展社会民生事业，人民群众有了更多获得感。坚持把市级新增财力的75%、县级新增财力的60%以上投入民生。每年办好十件民生实事，五年累计民生支出1088亿元，年均增长27.7%。居民人均可支配收入年均增长12.2%，城乡居民收入比由2.6∶1缩小为1.9∶1。获评全国首批民生改善典范市，连续六次获评“中国最具幸福感城市”。就业和社会保障体系日益完善。新增城镇就业34万人、创业2.55万人、转移农村劳动力7万人，城镇登记失业率控制在2.5%以内。城乡居民基本医疗保险实现全覆盖。企业退休人员月人均养老金增长99.1%。最低生活保障、农村“五保”供养、孤儿和城镇“三无”人员保障标准达到全省先进水平。全面免除殡葬基本服务费用。改造农村危房18832户，建设保障性住房33482套，解决24148户家庭住房困难问题。教育文体事业优质发展。新增普通中小学学位6.7万个，获评省推进教育现代化先进市，全国义务教育发展基本均衡县区实现全覆盖。惠州学院人才培养和科研学科建设取得重大进步。职教新城、技师学院投入使用，卫生职业技术学院、城市职业学院申办成功。引进广州珠江职业技术学院落户博罗。建成4所标准化特殊学校。国家、省公共文化服务体系示范项目及示范区创建顺利推进，发放文化惠民卡14.2万张。14个县级、73个镇级和1249个村级文化设施实现达标升级。市数字图书馆上线运行。文化遗产保护成效显著，新增省级“文保”单位17处、“非遗”名录8项，获评省历史文化街区5条。龙门县、惠东平海镇被评为“中国民间文化艺术之乡”。成功举办一大批文化体育赛事活动，参加省十四届体育运动会取得优异成绩。乡镇农民健身工程实现全覆盖。卫计事业加快发展。全面完成基层医疗卫生机构综合改革、县级公立医院改革任务，建成市中医医院新院、中大惠亚医院和广州中医药大学第三附属医院惠博医院，完成150间村卫生站标准化建设。有效防控H7N9禽流感、登革热等疫情，成功应对全国首例输入性中东呼吸综合征。获评全国首批创建幸福家庭活动示范市，人口与计划生育工作考核连续两年全省第一。文明创建实现“满堂红”。博罗、龙门入选全国文明城市（县级）提名城市，惠东获评省文明县城，新增全国文明单位5个、全国文明村镇3个，建成道德讲堂800个、乡村（城市）学校少年宫209所，注册志愿者超41万人，涌现中国好人12人、省级以上道德模范及身边好人43人。平安创建卓有成效。矛盾纠纷排查调处机制进一步健全，综治信访维稳网络加快完善。“雷霆扫毒”“6+1”“3+2”专项综合考核排名全省第一，禁毒百城会战获全国地级市第一名，禁毒工作经验全国推广。连续四次获评中国最安全城市，连续两次获评全国社会管理综合治理优秀市。食品药品监管进一步强化。建成全国首个国家级危险化学品应急救援基地。安全生产责任和管理制度不断完善，安全生产形势总体平稳。各项社会事业协调发展。获评全国首批法治城市创建先进市、全国“五五”普法先进城市、全国“六五”普法中期先进城市。成为《立法法》修订后全省第一批具有地方立法权的市。荣获全国双拥模范城“四连冠”，通过全国双拥模范城“五连冠”考评，所有县区成为省级双拥模范城。顺利通过国家卫生城市复审。市社会福利服务中心一期项目投入使用。建成居家养老服务中心19家、农村幸福院97个。对外合作水平不断提升，与澳大利亚、韩国、以色列、乌克兰、萨摩亚等国家合作务实推进。对口支援四川丹巴县、道孚县工作进展顺利。国防动员、人民防空、老区建设等工作取得新成绩。妇女儿童、青少年、老年人、残疾人、红十字等事业取得新发展。台务、口岸、民族宗教等工作取得新成效。统计、物价、供销、粮食、移民、气象、三防、消防、应急、防震、科普、档案、方志等工作取得新进步。

　　各位代表，五年来，我们始终把建设人民满意政府作为努力方向，不断加强法治政府、廉洁政府和服务型政府建设。坚持向市人大及其常委会报告工作，向市政协通报情况，主动听取各民主党派、人民团体和社会各界的意见和建议。五年办理市人大代表议案62件、建议750件，政协委员提案838件，办理答复率、征询意见率均达100%。坚持依法行政，建立健全重大决策征求意见、专家评估、合法性审查、集体决定制度。成立市政府决策咨询委员会，建立首批决策咨询专家库。市、县两级建立健全16个类别367项行政执法责任制度。依申请公开政府信息答复率100%。广泛开展“万众评公务”“行风热线”“惠民在线”等活动，行政效能明显提升。认真落实中央八项规定精神和国务院“约法三章”要求，深入开展党的群众路线教育实践活动和“三严三实”专题教育，切实加强“四风”整治。市直党政机关“三公”经费支出连续五年下降。监察工作扎实有效，审计全覆盖深入推进，政府廉政建设进一步强化。

　　各位代表，“十二五”时期是我市攻坚克难、综合实力大提升的五年，是改革创新、发展环境大优化的五年，是共建共享、民生质量大改善的五年。这五年所取得的成绩，是省委、省政府和市委坚强领导的结果，是在历届市领导班子打下的坚实基础上，全市人民继往开来、团结拼搏的结果。在此，我代表市人民政府，向为惠州建设作出重大贡献的老领导、老同志致以崇高敬意！向全市人民，向中央和省驻惠单位、驻惠人民解放军和武警官兵，向人大代表、政协委员、各民主党派、人民团体、社会各界人士，向长期关心支持我市改革发展的港澳同胞、台湾同胞、海外侨胞及国际友人，表示衷心感谢！

　　各位代表，五年的奋斗历程步履坚实，五年的奋斗成果来之不易，五年的奋斗经验弥足珍贵。回顾五年的实践，我们深刻认识到，向着以更好质量更高水平进入珠三角第二梯队目标迈进，必须始终坚持发展第一要务，着力推动经济发展提质增效；必须始终坚持改革创新，着力破阻力、激活力、增红利；必须始终坚持生态优先，着力增强绿色发展新优势；必须始终坚持发展惠民，着力建设本地人有自豪感、外地人有归属感的美好家园；必须始终坚持改进作风，着力营造为民、务实、清廉、高效的政务环境。

　　在总结成绩的同时，我们也清醒地认识到，我市经济社会发展还存在不少问题：一是经济总量还不够大，稳增长面临诸多压力，尤其是去年出口出现多年来未有的负增长。二是经济质量和效益有待提升，发展方式仍显粗放，单位土地产出效益偏低。三是产业结构过于单一，外贸依存度仍然偏高，经济抗风险能力较弱，结构调整任重道远。四是科技创新能力不强，高新技术企业、新型研发机构等在数量和质量上都与发达地区差距较大。五是区域发展不平衡、不协调问题依然存在，新型城镇化推进力度有待加大。六是协调各方利益、维护社会稳定压力较大，食品安全、公共安全和安全生产隐患不容忽视，创新社会治理的任务十分繁重。七是社会民生事业还存在薄弱环节，与人民群众期盼仍有差距。八是政府职能转变还不到位，行政效率有待提高，进一步解放思想尤为迫切。对于这些问题，我们将本着对人民高度负责的精神，坚持从严从实，采取有力措施切实加以解决。

　　“十三五”时期的奋斗目标和主要任务

　　“十三五”时期，是我市率先全面建成小康社会、以更好质量更高水平进入珠三角第二梯队的决胜阶段。综观国内外形势，世界经济在深度调整中曲折复苏，我国经济长期向好的基本面没有变，我省转型发展的前进态势没有变，我市经过多年积累和深化改革，发展基础更加厚实，特别是省委、省政府大力支持我市以更好质量更高水平进入珠三角第二梯队，一大批重大基础设施项目在我市布点落地，一大批重大产业项目加快建设，为我市加快发展注入了强劲动力。总的看，“十三五”时期，我市仍处于跨越发展的“黄金期”。我们必须正确处理好好与快、内与外、破与立、大与小、远与近等重大关系，深刻认识、主动适应、积极引领新常态，抢抓发展机遇，保持发展定力，有效化解各种矛盾，努力把各项工作做得更好，奋力开创率先全面建成小康社会、以更好质量更高水平进入珠三角第二梯队新局面。

　　“十三五”时期政府工作指导思想是：全面贯彻党的十八大和十八届三中、四中、五中全会精神，深入学习贯彻习近平总书记系列重要讲话精神，按照“四个全面”战略布局，践行创新、协调、绿色、开放、共享发展理念，落实省委十一届五次全会和市委十届九次全会、市第十次党代会第五次会议部署，坚持稳中求进、好中求快、改革创新、率先跨越，牢牢把握“四个发展核心”，补齐“五个发展短板”，增创“四个发展优势”，推动“五位一体”协调发展，确保率先全面建成小康社会、以更好质量更高水平进入珠三角第二梯队，为全省实现“三个定位、两个率先”目标多作贡献。

　　“十三五”时期我市经济社会发展主要目标是 “一个率先、三好四新”。“一个率先”就是率先全面建成小康社会。确立2017年为我市率先全面建成小康社会目标年，力争提前实现地区生产总值和城乡居民人均收入比2010年翻一番。“三好四新”就是经济发展质量更好、人民生活质量更好、生态环境质量更好，经济实力迈上新水平、创新驱动能力迈上新水平、城乡区域协调发展迈上新水平、社会和谐迈上新水平，以更好质量更高水平进入珠三角第二梯队。

　　做好“十三五”工作，实现“十三五”目标，必须把“五大发展理念”贯穿始终，围绕“四个发展核心”谋篇布局，针对“五个发展短板”发力使劲，重点做到以下七个方面：

　　——— 突出发展第一要务，推动经济发展实现新跨越。以提高发展质量和效益为中心，扩大有效供给，满足有效需求，规划建设300宗、总投资9669.8亿元的重大项目，带动形成1.38万亿元的固定资产投资规模，坚持投资消费出口协调拉动经济增长，实现地区生产总值年均增长9.5%左右，突破5000亿元；人均GDP年均增长8.5%左右，突破10万元。

　　——— 突出创新驱动，加快建设创新型城市。把创新摆在发展全局的核心位置，以科技创新引领全面创新，培育发展新动力，拓展发展新空间，力争到2020年全市高新技术企业1000家、新型研发机构20家、市级以上孵化器35家，全社会研发经费支出占地区生产总值的比重达3%，技术自给率和科技进步贡献率分别达75%、60%，加快形成以创新为主要引领和支撑的经济体系和发展模式，建成国家创新型城市。

　　——— 突出结构优化，促进产业向中高端发展。着力提高全要素生产率，加快形成质量、品牌、服务为核心的竞争优势，提升产业发展层次。大力推进结构调整，在做强做优石油化工、电子信息两大支柱产业的同时，推动汽车与装备制造、清洁能源成为新的支柱产业，大力发展金融、物流、旅游、电子商务、健康养生等现代服务业，加快发展海洋经济，积极培育物联网、云计算、LED、生物医药等新的优势产业，构建支柱多元、结构优化、支撑力强的“2+2+N”现代产业体系。

　　——— 突出统筹协调，提升城乡均衡发展水平。加快建设以“两港三网”为重点的基础设施，夯实区域协调发展基础。争取到2020年，高快速公路总里程达1000公里，实现所有乡镇30分钟内上高速公路；加快建设赣深高铁和广汕铁路等轨道交通，融入广州、深圳乃至香港“半小时生活圈”；完善宽带信息基础设施，建成无线城市；惠州港年吞吐能力达1.3亿吨；惠州机场旅客年吞吐量达150万人次。加快新型城镇化建设，到2020年，常住人口城镇化率达75%，实现城镇基本公共服务常住人口全覆盖，形成均衡协调发展新格局。

　　——— 突出绿色发展，建成国家生态文明建设示范市。始终坚持生态优先，加快建设资源节约型、环境友好型社会，单位地区生产总值能耗、二氧化碳排放、主要污染物排放总量完成省下达任务，城镇生活污水集中处理率达97%，城镇生活垃圾无害化处理率保持100%，森林覆盖率达62.7%，空气质量保持全国前列，着力打造天蓝、地绿、水净的美丽惠州，让我们的家园山清水秀、鸟语花香，让市民享受更多的“绿色福利”。

　　——— 突出改革开放，增强发展动力活力。主动对接“一带一路”战略和广东自贸区建设，加快融入全球化步伐，构建起全方位开放合作新格局，增创竞争新优势。坚持以开放促改革、促发展，推进供给侧结构性改革，全面深化各领域改革，率先在重要领域和关键环节改革上取得决定性成果，打造市场化法治化国际化便利化营商环境。

　　——— 突出共建共享，建设民生幸福城市。着力增进民生福祉，努力实现城乡居民收入增长与经济发展同步，到2017年全面完成脱贫攻坚任务，实现全国文明城市 “四连冠”，2019年获得全国双拥模范城 “六连冠”，到2020年居民人均可支配收入达到4万元。大力发展各项社会事业，完善社会保障体系，实施“健康惠州”工程，加快建设卫生强市，全面提升基本公共服务均等化水平，让发展成果更多惠及市民。

　　2016年工作安排

　　2016年，既是实施“十三五”规划的开局之年，也是我市率先全面建成小康社会的关键一年，做好各项工作意义重大。今年经济社会发展的主要预期目标是：地区生产总值增长9%～9.5%；人均GDP增长8%～8.5%；固定资产投资增长15%以上；外贸出口总额增长1%；社会消费品零售总额增长10.5%；地方一般公共预算收入增长10%以上；服务业增加值占地区生产总值比重40%左右；全社会研发经费支出占地区生产总值比重2.5%；居民人均可支配收入增长13%；居民消费价格指数控制在103%以内；城镇登记失业率控制在2.6%以内；人口自然增长率、节能减排等各项指标完成省下达任务。

　　实现全年目标，重点抓好以下九个方面的工作：

　　（一）全力稳定经济增长

　　扎实开展 “项目建设提速年”活动。把项目建设作为经济建设主战场，加强项目储备，实施项目库管理，破解项目落地瓶颈，完善项目推进机制，强化项目督导考核，推动项目谋划提速、开工提速、建设提速、达产提速。完成214宗重点项目590.2亿元年度投资计划，进一步提高2013年以来新立项亿元以上项目开工率和投产率。启动河惠莞高速、汕湛高速河清段、惠河高速白石互通建设，继续推进武深高速新丰至博罗段建设，确保建成潮惠高速惠州段、从莞高速惠州段。做好广汕铁路、赣深高铁惠州段启动工作和深惠城轨前期工作。坚持大招商招大商，强化与世界500强企业、跨国集团、大型央企省企务实合作，重点引进一批产业带动强、科技含量高、税收贡献大的优质项目。

　　积极扩大消费需求。着力稳住汽车和住房等大宗消费，落实小排量汽车、新能源汽车税收优惠等政策。加强与阿里巴巴战略合作，推动各县区建设农村淘宝县级服务中心、村级服务站，探索鞋业、服装等传统制造业与电子商务深度融合。持续推进惠货全国行、网上行，办好惠州产品南昌展销会，深入开展“消费促进月”等各种促销活动。继续抓好旅游宣传推介，落实带薪休假制度，推动旅游消费。扩大信息、教育、文化、体育等消费，加快拓展消费新空间。

　　加大企业帮扶力度。坚持亲商稳商，深化“企业服务月”活动，积极为企业排忧解难。深入实施 “企业成长计划”和壮大发展民营经济新 “惠42条”，加大财政扶持力度，培育企业梯队发展。继续实施经济户口清理，推动220家以上小型企业上规模。完善信贷风险补偿机制，加快建设市中小微企业融资服务平台，与省平台实现对接。鼓励设立股权投资企业，组建市中小微企业政策性担保公司。推广应用PPP模式，支持民营企业参与重大基础设施和公用事业项目，大力促进民营经济发展。

　　壮大财税金融实力。积极培植税源，坚持依法征税，确保财政收入稳定增长。加大盘活财政存量资金力度，优化财政支出结构，将更多财力向民生、基层倾斜。强化财政资金使用绩效考核，提高财政支出效率。大力发展银行、证券、保险产业。鼓励引导企业通过上市、发债和股权挂牌融资，推动1家以上企业上市和10家以上企业在新三板、区域股权市场挂牌。

　　加强经济运行监测。继续坚持经济运行监测月度例会和联合督导制度，加强对经济运行形势的分析和预判，确保年度各项目标任务顺利完成。加强市场价格监管，有效稳定物价。防范金融风险，维护地方金融安全，加强地方政府债务限额管理。

　　（二）大力推进创新驱动发展

　　加快建设惠州珠三角国家自主创新示范区。研究制订惠州国家自主创新示范区 《发展规划》、《实施方案》及相关政策文件，明确发展定位、发展目标和责任分工。确定产业主攻方向，培育具有国际领先水平的创新型产业集群。大胆探索，先行先试，优化创新创业生态，充分发挥示范区的核心带动作用。

　　全力提升“一基地、双平台”建设水平。加快编制潼湖智慧区专项规划，抓紧启动科技金融新城和国际合作产业园（中韩科技园）两个起步区建设，不断集聚各类创新要素，打造生态型、智慧型创新发展基地。加快推进仲恺高新区东江、惠南产业园扩容提质，抓好LED产业园、德赛汽车电子产业基地建设。加快环大亚湾新区三大起步区建设步伐，推动大亚湾科创园争创国家级科技企业孵化器。积极引进总部经济、3D打印、工业机器人、科技研发等项目，将环大亚湾新区、仲恺高新区“两大平台”建设成高端产业集聚发展的主阵地，引领带动全市产业园区创新发展。

　　着力提高企业自主创新能力。大力实施移动互联、石油化工、新能源汽车、LED等重大科技专项，力争突破一批关键核心技术和行业共性技术。大力培育发展创新型企业，加快引进和培育一批高新技术企业，新增高新技术企业150家。实施孵化器倍增计划，大力发展民营孵化器，推广“异地成果、惠州孵化（加速）”的做法，引进以色列先进经验，构建中以联合孵化器新模式。继续推进工业转型升级攻坚战三年行动计划，推动350家规模以上工业企业实施技改，技改投资增长20%以上。

　　广泛集聚科技创新资源。推动中科院“两所两装置”建设，争取省市联合建设“互联网+”研究院、设立自然科学基金。加快推动北京化工大学惠州研究院和暨南大学惠州研究院建设，新型研发机构达8家。推动主营业务年收入5亿元以上企业普遍设立研发机构。主动引进深圳先进技术成果，积极承接优质产业溢出。深化同以色列、乌克兰、德国、韩国等国家的科技合作。推动政产学研协同创新，促进科技创新成果转化。

　　持续优化创新创业环境。大力推进科技体制改革，不断完善“1+6+N”政策体系。落实研发费用加计扣除、高新技术企业税收减免等优惠政策，改革科技专项资金配置方式，实施新型研发机构、孵化器产权分割等科技扶持措施，激发科技创新动力。深入实施知识产权战略，力争万人发明专利申请量和授权量分别增长20%、25%以上。加快推进专利质押融资及风险补偿工作，积极实施重点产业专利导航计划，开展企业知识产权贯标工作，加大知识产权保护力度。支持银行设立科技金融事业部、科技支行，推动金融与科技深度融合。深入实施“人才双十行动”，加快引进创新创业团队和科技领军人才；推进人才公寓、人才服务大楼建设，成立高层次人才“一站式”服务中心，帮助人才安居乐业。加快建设低成本、便利化、全要素、开放式的众创空间，形成“大众创业、万众创新”的新态势。

　　（三）突出抓好改革攻坚

　　推动供给侧改革。抓好去产能、去库存、去杠杆、降成本、补短板五大任务，制定实施“去、降、补”工作方案。实施差别化政策，提高环保、能耗和技术标准，推动部分相对落后产能有序转移或退出。开展降本增效专项行动，落实降低制度性交易成本、企业税费负担、电力价格、物流成本等政策。推进质量强市建设，走以质取胜和品牌发展道路，增加中高端产品和优质服务供给。引导企业开拓线上线下销售市场，加快去库存步伐。改善住房供给结构，大力发展住房租赁市场，推进住房公积金省内异地互贷，促进房地产业健康发展。

　　深化行政体制改革。完成市、县政府部门权责清单确认公布。深入推进行政审批制度改革，进一步精简行政许可事项，加快推进行政许可标准化，推行 “一门式”“一网式”政府服务模式。实施投资项目统一编码制度。狠抓项目落地建设的全流程改革，压缩项目审批时间，推进项目投资建设直接落地。抓好市场准入负面清单制度改革。清理和规范职业资格许可认定。开展涉企收费项目清理，编制涉企收费目录清单。全面实施“三证合一”登记制度改革，推进电子营业执照和企业登记全程电子化改革。进一步加强行政服务中心、网上办事大厅建设，不断提高政务服务水平。

　　深化财税金融体制改革。探索实施跨年度预算平衡机制和中期财政规划管理，进一步完善预算管理制度。推进国地税征管体制改革，落实营改增扩面等税改工作。积极培育地方金融组织，扎实开展农村普惠金融服务工作。扩大政策性农业保险覆盖面。深化公共资源交易体制改革，抓好中介超市规范运作。

　　统筹抓好改革试点工作。全力推进20项国家级和54项省级改革试点。深化发展环境体系、社会治理体系等改革，发挥系统改革的整体带动效应。持续推进重点领域改革攻坚，争取在国企国资、社会保险等领域改革中取得新突破。深入推进社会信用体系建设，努力创建国家社会信用体系建设示范市。

　　（四）努力构建现代产业体系

　　发展壮大石化、电子两大支柱产业。全力推进中海油惠炼二期建设，确保按时投产。进一步扩大与壳牌集团等国际化工巨头合作，推进SMPO等石化中下游项目建设，完善提升石化园区配套服务，加快建设世界级石化产业基地。大力发展移动终端、平板显示、LED、新能源电池等高端新型电子信息产业，确保信利AMOLED一期、伯恩光学玻璃生产基地顺利投产，全力推进信利二期项目，支持TCL和三星做大做强，巩固发展国家级电子信息产业基地。办好2016中国手机创新周和第五届云博会，加快打造"世界手机之都"。

　　着力培育新的支柱产业。大力发展战略性新兴产业，改造提升传统产业，推动汽车与装备制造、清洁能源成为新的支柱产业。编制实施先进装备制造业发展规划，着力发展 "工作母机"类制造业，培育装备制造骨干企业，成立先进装备制造业产业联盟。突出发展新能源汽车整车与关键零部件产业，加快推进博罗、大亚湾汽车产业园和惠东京兰新能源汽车等项目建设，推动比亚迪、德赛、华阳、亿纬锂能等汽车重点项目扩大产能。积极发展核电、风电、太阳能、LNG发电和蓄能水电等清洁能源产业，扎实推进广东太平岭核电、惠州LNG接收站等项目建设。

　　大力发展现代服务业。以生产性服务业为重点，大力发展科技服务、工业设计和文化创意等新业态，培育壮大一批生产性服务业龙头企业。加快发展空港经济，推进惠州机场二期改扩建，逐步增加航班线路和密度，争取惠州机场航空口岸纳入国家口岸发展规划。推动华瀛30万吨原油码头、深能源煤码头、惠州港引航基地等项目建设，加快建设亿吨大港。规划建设罗浮山至南昆山百里生态旅游长廊、环大亚湾百里滨海休闲长廊，加快推进惠州西湖创5A景区、巽寮湾创国家级旅游度假区，提升罗浮山、环南昆山等景区建设水平。整合旅游资源，打造旅游精品线路，推动博罗、龙门全域旅游示范县建设，争创国家全域旅游示范市。积极参与中国铁路国际物流中心建设，大力发展现代物流业。加快发展健康养生、信息服务等新兴服务业。

　　（五）积极推进外经贸转型升级

　　全力稳定外贸增长。组织企业参加境内外重点展会，办好惠州产品印度展览会。着力发展外贸新业态，大力培育引进外贸综合服务龙头企业，加快建设跨境电商产业园及仓储物流中心。用足用活国家政策，积极申报外经贸专项扶持资金。扩大先进技术设备、大宗商品、优质消费品等进口。加快通关制度改革，提高口岸通关效率和贸易便利化水平。

　　加快外经贸转型发展。鼓励加工贸易企业扩大研发投入，加强品牌建设，推动转型升级。落实好培育壮大一般贸易政策措施，大力发展一般贸易。促进服务贸易领域对外开放，加快发展服务外包。支持有实力的企业扩大对外投资。提升仲恺高新区省级外贸转型升级示范基地、TCL液晶产业国家科技兴贸创新基地建设水平。积极运用广东自贸区制度创新成果，促进外贸转型发展。

　　深化对外交流合作。积极参与粤港澳大湾区建设，深化与港澳台经贸、科技、文化、医疗等多领域交流合作。加强与欧美发达国家、金砖国家和日本、韩国等国家（地区）的多层次经贸合作，提升外资利用水平。积极参与"一带一路"建设，巩固东盟市场，大力拓展古巴、墨西哥等北美市场以及南亚、非洲等新兴市场。积极争取澳洲肉牛进口项目落户，扎实推进与南太平洋岛国务实合作。

　　（六）加快推进新型城镇化

　　抓好国家新型城镇化综合试点。推进户籍制度改革，有序放开落户条件，促进农业转移人口市民化，鼓励务工人员就近购房，逐步实现基本公共服务常住人口全覆盖。坚持以工促农、以城带乡，推动教育、文化、医疗等资源向镇村延伸，促进城乡公共资源均衡配置，以基本公共服务均等化促进城乡区域协调发展。

　　提升城市建管水平。改革完善城市规划管理体制，推进多规合一、多规融合，进一步增强规划的强制性和严肃性。贯彻"适用、经济、绿色、美观"的建筑方针，加快市民公园、美术馆、城市规划馆等建设，打造城市建设新亮点。抓好水电路气、地下管廊、防洪排涝等市政工程，建设"海绵城市"。有序推进"三旧"、城中村、棚户区和城乡结合部集中联片改造。加快城市管理和行政执法体制改革，保持"两违"整治高压态势。按照"一市六组团"布局，加快推进县城扩容提质，提升县城产业发展和公共服务承载能力。

　　改善交通出行条件。加快隆生大桥、新开河北桥、龙桥大桥、龙江大桥、芦泰东江大桥建设，继续推进仲恺大道、惠新大道、四环路南段、金龙大道和市区西出口改造等工程。抓好市汽车客运南站、市中心客运枢纽和龙门、博罗客运中心站建设。实施市区公交专用道建设，做好公交线路与城轨、高铁、机场对接。新增及优化16条公交线路，新增或更新160辆公交车，进一步完善公交线网。新投放2000辆惠民自行车。整治超标电动车。积极创建省级公共交通示范城市。完成国道205线、省道119线改造，加快国道324线惠东陈塘至小屯段、省道120线、省道355线等改扩建。

　　推进电力、信息基础设施建设。全力抓好国家智慧城市试点和国家信息消费试点城市工作，大力建设光纤网络、4G通信网络、公共区域免费WiFi等信息基础设施，加快建设信息高速公路，完善宽带信息网，建设无线城市。完善"数字市政"系统，发挥数字城管平台作用，提升城市管理信息化水平。加快推进500千伏崇文输变电工程，完成新一轮农村电网改造升级任务。

　　积极参与深莞惠经济圈 （3+2）建设。深入实施珠三角改革发展规划纲要，全方位对标深圳，加强与周边城市基础设施互通、产业合作互补、创新资源互享、污染治理互助、社会管理互动，提升区域合作水平。

　　（七）扎实做好农业农村工作

　　完善农村基础设施。以规划为引领，实现村庄规划全覆盖，提高村镇建设水平。完成新农村公路建设800公里、县道改造20公里。抓好农村公路危桥改造。出台村村通自来水工程实施方案，确保年内57个贫困村自来水进村到户。扎实抓好博罗、惠阳、惠东三个省级新农村示范片项目，带动全市新农村建设。继续推进名镇名村建设，创建名镇2个、名村20个。

　　加快现代农业发展。加强耕地保护，完成永久基本农田划定工作，建设高标准农田16.1万亩、现代农业综合示范区6个。"十二大"特色农产品种植面积稳定在210万亩以上。深入推进农业科技化、机械化，农业科技贡献率、机械化水平分别达到64%、72.3%。新增市级以上农业龙头企业10家、市级农民专业合作社示范社20家。大力发展休闲农业，拓宽农民增收渠道。推进现代渔港建设。做好第三次全国农业普查工作。

　　深化农业农村改革。加快农村土地承包经营权确权登记颁证工作，深入推进农村集体产权制度改革，加大农村资产监管力度。稳妥推进集体经营性建设用地入市、农村承包土地经营权和农民住房财产权抵押贷款。抓好市属国有林场改革。支持博罗开展全国低丘缓坡地综合利用开发试点工作。

　　大力实施精准扶贫。按照"六个精准""五个一批"的要求，科学制定新时期精准扶贫、精准脱贫实施方案，全面实施脱贫攻坚工程，突出抓好少数民族地区和革命老区扶贫开发，采取"一村一策、一户一法"，实行定点、定人、定责帮扶，强化考核，推动46个省重点帮扶村、18138户相对贫困户、41784名相对贫困人口脱贫。

　　（八）持续加强生态文明建设

　　强化生态创建。严格执行生态低碳规划，落实主体功能区分区管控，加强生态控制线划定管理，重点生态功能区实行产业准入负面清单制度。开展新一轮森林资源二类调查，深入实施林业四大重点工程，扎实推进新一轮绿化惠州大行动，建设生态景观林带86公里，新建森林公园2个、湿地公园1个，继续抓好市植物园、潼湖湿地公园、海洋生态园建设。加强海岸带保护利用，扎实推进考洲洋美丽海湾、东山海人工鱼礁示范区2个省重点海洋生态整治修复项目建设。继续开展 "美丽乡村·绿满家园"行动，高标准建设森林村庄120个。加快创建国家生态文明建设示范市，力争创建国家级生态乡镇40个、省级生态县区2个。

　　强化污染防治。深入推进全国水生态文明城市建设试点，继续实施"美丽乡村·清水治污"行动，加快建设金山二期、梅湖三期、马安污水处理厂，抓好截污管网建设和污水处理厂提标升级，新建200座以上农村污水处理设施，提高城镇生活污水收集处理率。继续推进淡水河和潼湖流域污染治理，大力推进望江沥、新开河、大湖溪沥、洛塘渠以及63条镇级河涌污染整治。加强东江和西枝江饮用水源保护。继续深化"美丽乡村·清洁先行"行动，扎实做好城乡生活垃圾分类工作，建成惠阳垃圾焚烧发电厂，推进市生态环境园和惠东、龙门垃圾焚烧发电厂建设，提升垃圾综合处置能力。

　　强化节能减排。抓好省碳普惠制试点；实施重点节能工程，推广节能低碳技术，推动工业、建筑、交通运输等领域节能减排；实施新能源汽车推广计划，提高新能源公交车比例，完成省下达的污染减排任务。深入开展大气环境整治"八项行动"，保持良好空气质量。扎实开展大亚湾海域排污总量控制制度试点，保持近岸海域水质优良。

　　（九）全面发展社会民生事业

　　积极建设健康惠州。制订出台建设卫生强市实施方案。加快推进市中心人民医院总部建设，高标准规划建设健康城。积极推进市第一人民医院二期工程、特殊人员收戒羁留医院建设，规划迁建市第二人民医院，启动市皮肤病医院建设和复退军人医院改扩建工程。继续抓好379间村卫生站标准化建设，推动优质医疗卫生资源向基层下沉，提升基层医疗卫生服务能力。抓好城市公立医院改革试点，构建分级诊疗服务模式。按照省级标准建立1～3个临床医疗科研中心，力争建立1家网络医院。推广"智慧医疗"试点工作，基本完成二级以上医院信息化建设。大力发展中医药事业，办好第三届中医科学大会。

　　全面推进教育现代化。优化教育资源配置，提升办学整体质量。推进仲恺义务教育教师队伍"县管校聘"管理改革。完善异地务工人员随迁子女积分入学管理机制，做好随迁子女在当地参加中考高考工作。提高公办幼儿园和普惠性幼儿园比例；完善幼儿园收费定价机制，促进农村幼儿园保教水平和学前教育质量提升。加大民办学校扶持力度，建立民办学校教师长期从教津贴补助机制。逐步推进高中阶段免费教育。支持惠州学院加快发展，支持惠州经济职业技术学院升格为本科院校，积极申办惠州工程技术职业学院。推动国际教育合作，加快筹建新的理工科大学。

　　加强就业和社会保障。推进就业孵化基地建设，争创创业型城市，扶持创业5000人，带动就业1.7万人，新增城镇就业人员7万人、农村富余劳动力转移就业8000人。预防处置劳资纠纷，推进大亚湾和谐劳动关系综合试验区建设，积极构建和谐劳动关系。加快推进城乡居民社保一体化，深入实施全民参保计划。推进机关事业单位养老保险制度改革。认真做好城镇职工养老保险扩面征缴工作。加大住房保障力度，改造城市棚户区1550户、农村危房6000户。推进市社会福利服务中心二期工程建设，完成各县区省二级以上养老机构建设。加快推进社区综合服务中心和"一站三中心"建设。

　　大力发展文体事业。编制惠州古城文物古迹连片保护规划，重修东坡祠，修缮宾兴馆，抓好葛洪博物馆、博罗银岗古窑遗址公园等项目建设，提升文物单位和"非遗"名录项目保护级别，推进文化遗产保护立法工作。完成省公共文化服务体系标准化试点、示范区创建和第二批国家公共文化服务体系示范项目的迎检验收工作。繁荣文化艺术，发展文化产业。广泛开展全民健身活动，办好第五届市运会。做好省足球试点城市申报工作。

　　巩固文明创建成果。深入实施巩固文明成果三年提升计划，全力开展核心价值观培育践行、道德先进典型选树等"德行惠州"十大行动，加快博罗、龙门、惠东创建文明城市步伐，推进"好人之城"建设，推动市民素质和城市文明程度不断提升。

　　深化平安惠州创建。推动"畅顺惠州""惠民110"服务名片升级和"五项建设"落实，重拳打击毒品犯罪、涉众型犯罪、"两抢一盗"等多发性犯罪，完善立体化、网格化治安防控体系。深入推进无毒惠州创建，扎实做好全国仲裁"两化"试点和反走私诚信体系建设试点。加强信访维稳工作，及时回应解决群众合理诉求。严格落实安全生产责任制，坚决遏制各类安全生产事故。按照"四个最严"的要求，建立健全食品安全治理体系，切实保障人民群众"舌尖上的安全"。加强药品监管和产品质量管理，打击走私贩私、制假售假、传销和变相传销，切实维护市场秩序。

　　统筹发展社会各项事业。编制"七五"普法规划和"法治惠州"五年规划，争创全国首批法治城市。依法实施全面两孩政策，健全优生优育服务体系，加强计划生育服务管理，保持全省先进。深入开展爱国卫生运动，巩固提升国家卫生城市创建成果，推动博罗、惠东、龙门创建国家卫生县城。继续抓好双拥工作，加强国防动员和后备力量建设，推进军民融合深度发展。扎实推进对口援藏援疆援川工作。积极发展妇女儿童、青少年、老年人、残疾人、红十字等事业，继续抓好防震减灾、老区建设、民族宗教、三防、消防、应急、粮食、统计、台务、方志、档案、气象、移民、科普等工作，推动社会各项事业全面进步。

　　与此同时，今年我们将投入86.9亿元办好十件69项民生实事，继续推进基本公共服务均等化，增强群众获得感。

　　各位代表，做好今年经济社会发展各项工作，必须加强政府自身建设，更好发挥政府作用。始终坚持解放思想。按照市第十次党代会第五次会议的决策部署，迅速掀起新一轮思想大解放热潮，全方位向深圳学习，全面对标深圳，以思想的解放冲破旧观念，以思想的解放谋划新举措，以思想的解放促进效率大提升。切实加强民主法治建设。自觉接受人大及其常委会的监督，认真落实其各项决议、决定并定期报告工作。积极支持人民政协履行政治协商、民主监督、参政议政职能，认真听取各民主党派、工商联、无党派人士和各人民团体的意见建议。办理好人大代表建议和政协提案。全面推进依法行政。坚持"法无授权不可为、法定职责必须为"，推进政府及其部门职责法定化。推进重大行政决策程序立法和合法性审查，建立重大决策终身责任追究制度及责任倒查机制。积极参与地方立法工作，规范政府规章制定。继续推行行政执法责任制，提升执法规范化水平。推进行政机关负责人出庭应诉工作。全面推进政务公开，让行政权力在阳光下运行。持续深化作风建设。切实增强政治意识、大局意识、核心意识、看齐意识，深入践行"三严三实"。强化责任担当，确保中央、省和市委的部署落到实处、取得实效。强化效能建设，全面整治慵政懒政怠政，认真解决"乱作为、不作为、慢作为"问题。着力抓好廉政建设。认真落实中央八项规定精神、国务院"约法三章"要求，全面落实政府系统党风廉政建设主体责任，加强行政监察和审计监督，强化廉政风险防控，严肃查处各类违纪违法案件，使广大公务人员心有所畏、言有所戒、行有所止，努力打造廉洁高效、务实为民、法治诚信的政府。

　　各位代表！"十二五"的辉煌成就令人鼓舞，"十三五"的宏伟目标催人奋进。百舸争流，破浪者领航；千帆竞发，奋勇者当先。让我们在省委、省政府和市委的坚强领导下，紧紧依靠全市人民，以更加饱满的热情、更加昂扬的斗志、更加务实的作风，解放思想，锐意进取，真抓实干，为实现率先全面建成小康社会、以更好质量更高水平进入珠三角第二梯队的目标而努力奋斗！
